# Paracentral Acute Middle Maculopathy in a Young-Onset Case Revealing Undiagnosed Hypertension

**DOI:** 10.7759/cureus.94903

**Published:** 2025-10-19

**Authors:** Shunsuke Tokui, Yoichiro Shinohara, Hideo Akiyama

**Affiliations:** 1 Department of Ophthalmology, Graduate School of Medicine, Gunma University, Maebashi, JPN

**Keywords:** hypertension, optical coherence tomography, optical coherence tomography angiography, paracentral acute middle maculopathy, retinal ischemia

## Abstract

Paracentral acute middle maculopathy (PAMM) is a rare retinal ischemic disorder resulting from ischemia of the intermediate and deep retinal capillary plexus (DCP), causing secondary injury to the inner nuclear layer (INL). It typically occurs in older adults with vascular risk factors but can rarely present in young patients without prior systemic disease. A 37-year-old man presented with sudden visual impairment in the right eye. Fundus photography and fluorescein angiography were unremarkable; however, Humphrey visual field testing revealed a paracentral scotoma. Optical coherence tomography (OCT) revealed a band-like hyperreflective lesion in the INL, and OCT angiography (OCTA) revealed a localized DCP dropout. Systemic evaluation identified previously undiagnosed hypertension (213/136 mmHg), and laboratory tests excluded diabetes, dyslipidemia, and hypercoagulable disorders. Therefore, oral antihypertensive therapy was initiated. Three months later, blood pressure normalized (127/86 mmHg), the intensity and thickness of the OCT lesion decreased, and visual symptoms improved, although DCP dropout persisted on OCTA. This case demonstrates that young-onset PAMM may be the first sign of systemic hypertension. Elevated blood pressure can induce retinal microvascular ischemia, and timely management can lead to partial functional recovery despite persistent structural changes. Comprehensive systemic evaluation is recommended in young patients with PAMM to prevent further ischemic damage and improve outcomes.

## Introduction

Paracentral acute middle maculopathy (PAMM) is characterized by a paracentral hyperreflective band in the middle retinal layers on optical coherence tomography (OCT), reflecting ischemic injury of the inner nuclear layer (INL) [1]. Intermediate retinal tissue in the watershed zone is vulnerable to hypoxia owing to inadequate blood supply from the terminal choroidal and retinal vessels. PAMM results from impaired perfusion in the deep retinal capillary plexus (DCP) and intermediate retinal capillary plexus, leading to ischemia of the INL, and is often associated with permanent paracentral scotoma [2]. PAMM may occur as an isolated condition or in association with various retinal vascular diseases, including retinal artery occlusion [3], retinal vein occlusion [4], and diabetic retinopathy [5]. Moreover, systemic hypertension may induce PAMM due to retinal microvascular damage [6]. Previous studies have suggested that PAMM is more commonly observed in older males with systemic comorbidities, while its occurrence in young patients without a prior medical history is relatively rare [7]. Herein, we present the clinical course of a young-onset PAMM case, highlighting the OCT and OCT angiography (OCTA) findings before and after blood pressure management.

## Case presentation

**Figure 1:**
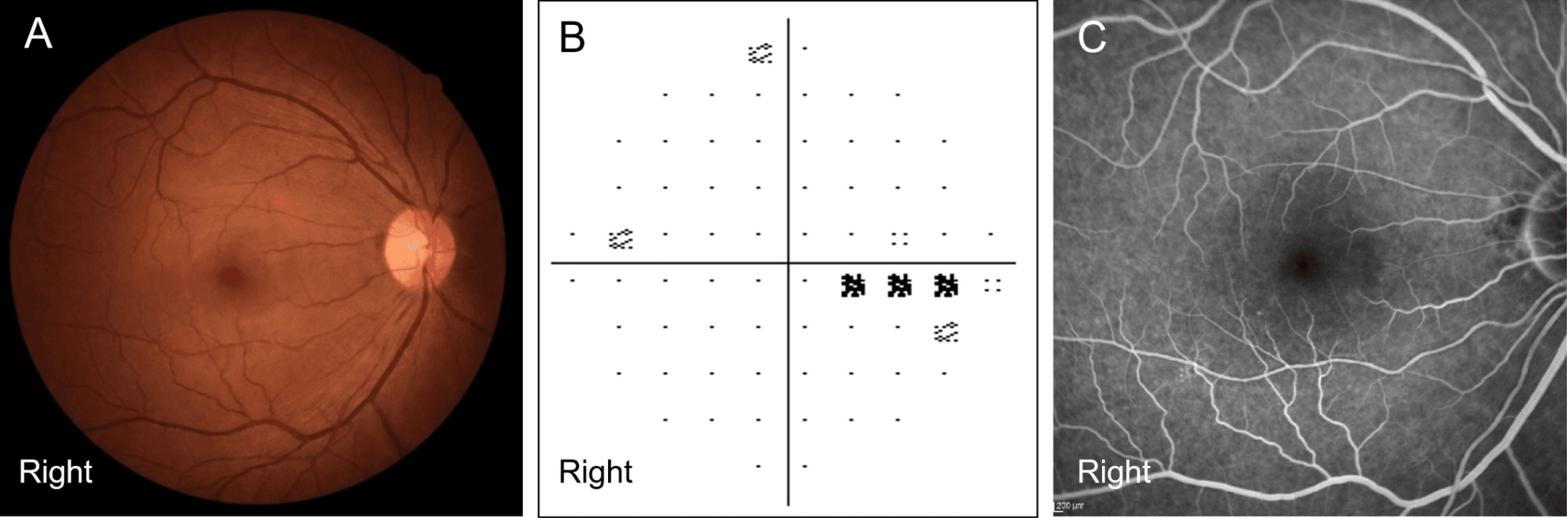
Ophthalmological findings on the first visit of a 37-year-old male patient. Best-corrected visual acuity in the right eye was 20/16. (A) No hypertensive changes were observed in the fundus of the right eye. (B) Humphrey visual field test (10-2) of the right eye, revealing a scotoma in the central temporal region, with the mean deviation of -1.82 dB. (C) Fluorescein angiography results are almost normal in the right eye.

A 37-year-old man was referred to our department because of sudden vision impairment in his right eye that began several days before presentation. The patient first noticed the symptoms when covering one eye, observing that the central visual field of his right eye appeared darker and slightly blurred compared to the left, which prompted him to seek medical attention. His medical history was unremarkable, although he had not undergone regular health checkups for several years. At the initial examination, the best-corrected visual acuity was 20/16 in both eyes, and the intraocular pressure was within normal limits. Slit-lamp examination and fundus photography revealed no abnormalities in either eye (Figure 1A). The Humphrey visual field test (10-2) demonstrated decreased sensitivity in the temporal central visual field of the right eye (Figure 1B). The patient's mean deviation (MD) was -1.82 dB. Fluorescein angiography showed no delayed filling or areas of nonperfusion in right eye (Figure 1C). The left eye demonstrated no abnormalities on fundus photography, Humphrey visual field test, or fluorescein angiography.

**Figure 2:**
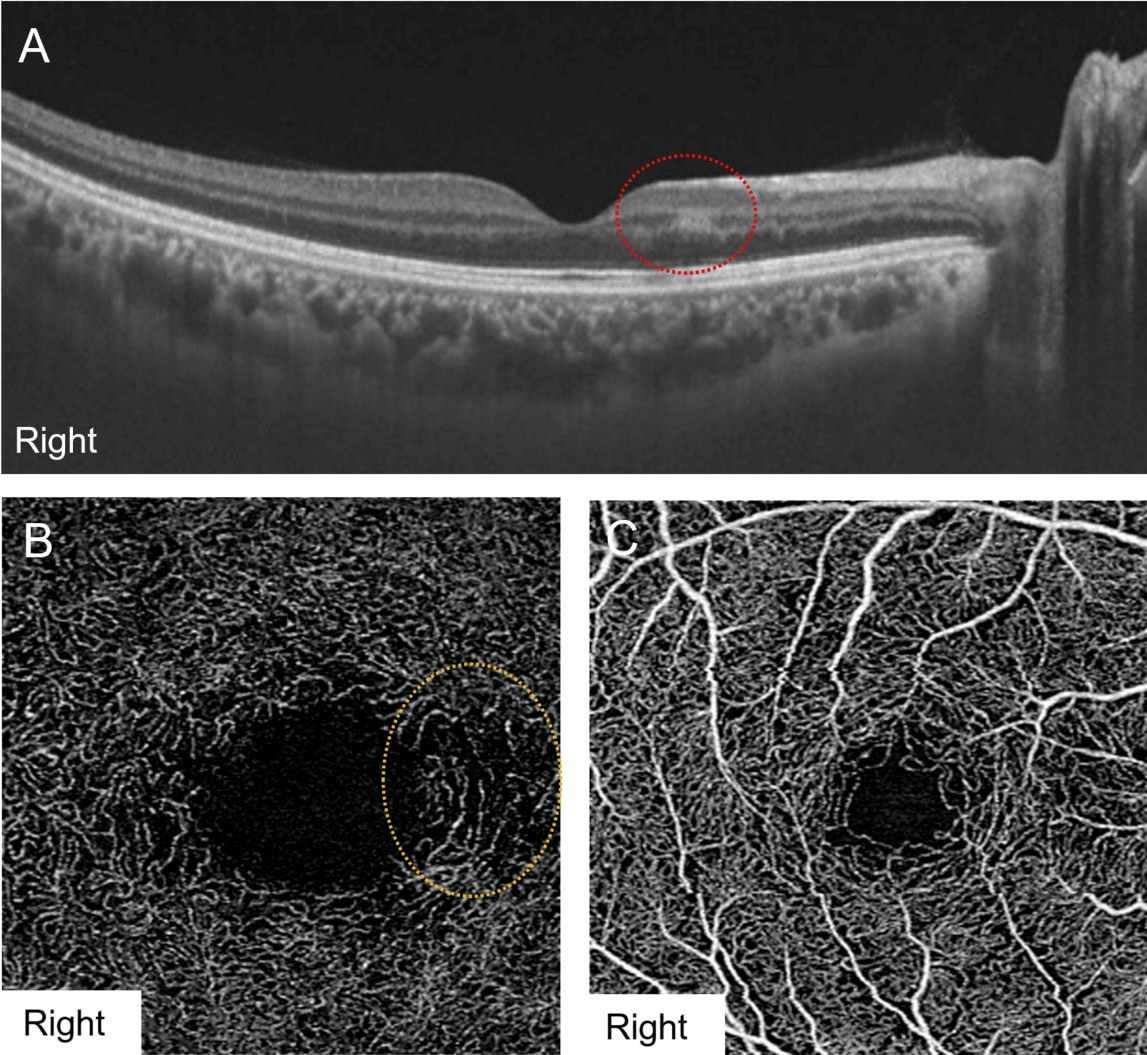
SS-OCT and OCTA findings at the first visit. (A) SS-OCT of the right eye showing a band-like hyperreflective lesion in the inner nuclear layer, consistent with paracentral acute middle maculopathy (red dotted circle). (B) OCTA of the deep retinal capillary plexus in the right eye, revealing localized capillary dropout in the paracentral nasal region (yellow dotted circle). (C) OCTA of the superficial retinal capillary plexus showing no capillary dropout in the right eye. SS-OCT: swept-source optical coherence tomography; OCTA: optical coherence tomography angiography

Swept-source optical coherence tomography (SS-OCT) (DRI OCT-1 Triton; Tokyo, Japan: Topcon Corporation) revealed a band-like hyperreflective lesion within the INL along the papillomacular bundle in the right eye, sparing the outer retinal layer (Figure 2A). The patient’s left eye was unremarkable. On the initial OCTA (PLEX Elite 9000; Jena, Germany: Carl Zeiss Meditec, Inc.), a localized loss of the DCP corresponding to the OCT hyperreflective lesion was observed (Figure 2B), whereas the superficial retinal capillary plexus (SCP) remained intact in the right eye (Figure 2C). To quantify the retinal vascular area in this patient, OCTA images were analyzed using ImageJ (Bethesda, MD: National Institutes of Health). A binarization algorithm was applied to differentiate vascular and interstitial regions, and the vascular area density (VAD) was calculated using the following formula: VAD(%)=vascular area/total area×100. At the initial visit, the deep VAD of the right eye was 35.43%, and the superficial VAD was 40.24%.

Although the patient was young and had no other health conditions, a systemic evaluation was performed for retinal vascular disease. Blood pressure was markedly elevated (213/136 mmHg). Laboratory investigations revealed no evidence of diabetes, dyslipidemia, or hypercoagulable disorders. Based on these findings, the patient was diagnosed with PAMM associated with elevated blood pressure. The patient was confirmed to have untreated systemic hypertension and was promptly started on oral antihypertensive therapy by the internal medicine department.

**Figure 3:**
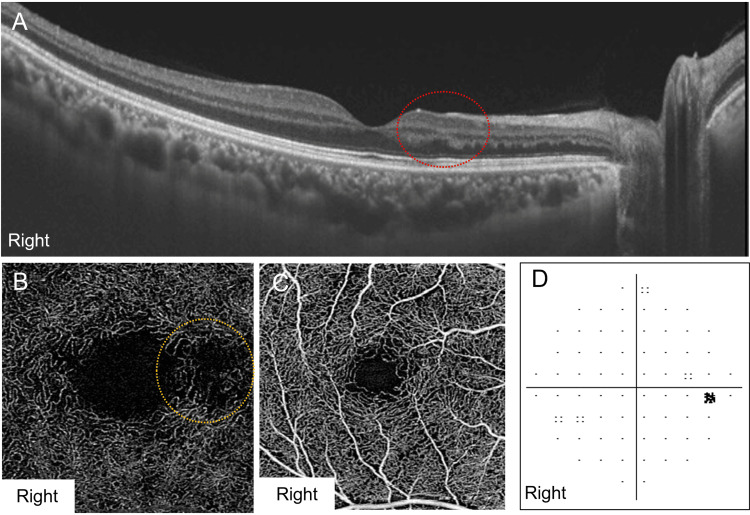
Ophthalmological findings three months after the initial visit. The best-corrected visual acuity was 20/16 in the right eye. (A) Swept-source optical coherence tomography of the right eye showing a hyperreflective lesion reduction and thinning in the inner nuclear layer, consistent with retinal ischemic perivascular lesions (red dotted circle). (B) Optical coherence tomography angiography (OCTA) image of the deep retinal capillary plexus in the right eye showing persistent capillary dropout (yellow dotted circle). (C) The OCTA image of the superficial retinal capillary plexus in the right eye showing normal findings. (D) Humphrey visual field test (10-2) of the right eye showing partial improvement in the central temporal scotoma, with the mean deviation improving to -0.88 dB.

Three months after initiating the antihypertensive therapy, his blood pressure decreased to 127/86 mmHg. SS-OCT of the right eye showed that the hyperreflective band in the INL became less intense and thinner than baseline (Figure 3A). DCP dropout persisted on OCTA, whereas SCP remained preserved (Figures 3B, 3C). The deep VAD of the right eye was 36.09%, and the superficial VAD was 40.60%. The patient’s visual complaints resolved, and the Humphrey visual field test (10-2) demonstrated partial recovery of the defect detected before treatment, with the MD improving to -0.88 dB (Figure 3D).

## Discussion

PAMM is a rare retinal disorder characterized by ischemic changes in the intermediate retinal layers, primarily the INL. PAMM is typically reported in older individuals with vascular risk factors, and its occurrence in younger patients without prior history is uncommon. In this case, a patient in his 30s presented with a paracentral scotoma caused by PAMM, and hypertension was incidentally discovered as an underlying condition. Notably, OCT after initiation of antihypertensive therapy revealed decreased hyperreflectivity and thinning of the INL, accompanied by substantial improvement in the patient’s visual symptoms.

PAMM is generally considered a microvascular ischemic disorder resulting from impaired perfusion of the DCP. The intermediate retinal layer functions as a watershed area with a low oxygen tension, rendering it particularly susceptible to ischemia [8]. OCT typically reveals hyperreflective lesions in the INL during the acute phase, which may progress to thinning or atrophy during the chronic phase. Focal PAMM may preserve retinal perfusion in the acute phase, whereas chronic PAMM is characterized by progressive loss of the DCP. In contrast, PAMM associated with severe ischemic conditions, such as central retinal artery occlusion, demonstrates substantial DCP loss in both acute and chronic stages [9]. In this patient, fluorescein angiography did not reveal any abnormalities, highlighting its limitations in visualizing the intermediate and DCP [10]. Therefore, OCT and OCTA are essential tools for diagnosing PAMM.

This case provides insight into the pathophysiology of retinal ischemia in young patients with previously undiagnosed hypertension; elevated blood pressure induces microvascular damage and may predispose them to PAMM. No significant difference in superficial retinal capillary plexus (SCP) and DCP vascular density was observed between the mild hypertension group and healthy controls. Nevertheless, chronic PAMM was reported in 88.9% of patients with mild hypertension and in 16.7% of healthy individuals [6]. Chronic PAMM can evolve into retinal ischemic perivascular lesions (RIPLs), which are characterized by permanent thinning of the INL and compensatory upward expansion of the outer nuclear layer, resulting in a wavy retinal architecture and persistent scotomas [11]. Notably, in this case, despite the presence of RIPLs, the paracentral scotoma improved. The absence of prior retinal disease, preservation of the ellipsoid zone, and the focal rather than diffuse nature of the PAMM lesions may have contributed to this functional recovery [12].

Most reports of PAMM in young patients describe an association with systemic diseases, such as sickle cell traits and primary antiphospholipid syndrome [13,14]. Other cases have been reported in healthy young individuals during pregnancy [15], migraine with aura [16], or coronavirus disease vaccination [17]. However, cases of incidentally discovered hypertension and functional recovery after blood pressure control are relatively rare. Improvement in blood flow in the acute phase, as observed in this patient, may have facilitated the partial restoration of retinal ischemia.

This case highlights the importance of a comprehensive systemic evaluation of young patients presenting with PAMM. Early detection and management of underlying risk factors, such as hypertension, may influence functional outcomes. PAMM in the absence of pre-existing retinal disease may indicate a serious systemic vascular disorder, warranting prompt investigation [18].

## Conclusions

Young-onset PAMM can serve as an initial manifestation of systemic vascular diseases, including previously undiagnosed hypertension. This case demonstrates that early recognition and treatment of hypertension can lead to a partial recovery of visual function, even in the presence of residual structural changes on OCT and OCTA. The presence of PAMM in young individuals may provide important diagnostic clues to underlying asymptomatic or latent hypertension.
